# Efficacité du traitement antirétroviral hautement actif seul dans le traitement du syndrome d'infiltration lymphocytaire diffus chez une patiente ivoirienne vivant avec le VIH : à propos d'un cas

**DOI:** 10.48327/mtsibulletin.2021.118

**Published:** 2021-08-20

**Authors:** I. Cherif, Y.M. Tsevi, L.D. Bawe, C. Guei, H. Yao

**Affiliations:** 1 Université de Cocody, Centre hospitalier universitaire de Yopougon, Abidjan, Cote d'Ivoire; 2 Université de Lomé, Centre hospitalier universitaire Sylvanus Olympio, Lomé, Togo; 3 Université de Cocody, Centre hospitalier universitaire de Treichville, Abidjan, Cote d'Ivoire

**Keywords:** VIH, DILS, Lamuvidine, Abacavir, Raltegravir Traitement antirétroviral, Hôpital, Abidjan, Côte d'Ivoire, Afrique subsaharienne, HIV, DILS, Lamuvidine, Abacavir, Raltegravir Antiretroviral treatment, Hospital, Abidjan, Ivory Coast, Sub-Saharan Africa

## Abstract

**Objectif:**

Nous rapportons dans ce travail l'efficacité des antirétroviraux (ARV) hautement actifs seuls dans le traitement d'un syndrome d'infiltrat lymphocytaire diffus (DILS) sans utilisation des corticoïdes qui parait risquée chez des patients vivants avec le VIH.

**Observation:**

Il s'agit d'une patiente de 60 ans séropositive au VIH, découvert au cours du bilan étiologique d'une insuffisance rénale qui retrouvait un profil glomérulaire non néphrotique. La ponction de biopsie rénale retrouvait un infiltrat interstitiel de CD8 évoquant un DILS. La prise en charge a consisté à la mise sous traitements ARV seul (lamuvidine, abacavir et raltegravir) sans corticothérapie associée. L’évolution clinique sous traitement a été marquée par une récupération de la fonction rénale avec une créatininémie à 99 μmol/l, une régression de la protéinurie, un taux de CD4 à 293/mm^3^ et une charge virale VIH à 533, 3 copies soit 1, 6 log en l'espace de 3 mois.

**Conclusion:**

Le DILS réalise une atteinte systémique diffuse chez les patients VIH ayant le plus souvent un contrôle virologique insatisfaisant. Devant la forte immunodépression et l'absence d'autres atteintes infiltratives, il nous est apparu risqué et injustifié d'adjoindre une corticothérapie.

## Abstract (en)

**Objective:**

We report in this work the efficacy of highly active antiretrovirals (ARVs) alone in the treatment of diffuse infiltrative lymphocytosis syndrome (DILS) without the use of corticosteroids, which appears risky in patients living with HIV.

**Observation:**

This is a 60-year-old HIV-positive patient, discovered during the etiological workup of renal failure, which revealed a non-nephrotic glomerular profile. The renal biopsy found an interstitial infiltrate of CD8 suggestive of DILS. Management consisted in starting ARV treatment alone (lamuvidine, abacavir and raltegravir) without associated corticosteroid therapy. The clinical evolution under treatment was marked by a recovery of the renal function with a creatininemia at 99 μmol/l, a regression of the proteinuria, a CD4 rate at 293/mm^3^ and an HIV viral load at 533.3 copies or 1.6 log in the space of 3 months.

**Conclusion:**

DILS is a diffuse systemic disease in HIV patients who are usually under poor virological control. In view of the strong immunosuppression and the absence of other infiltrative diseases, it appeared to us to be risky and unjustified to add a corticosteroid therapy.

## Introduction

Plus de 37, 6 millions de personne vivent dans le monde avec le VIH, dont 1, 5 millions sont nouvellement infectées [4]. En Afrique de l'ouest et du centre, plus de 4, 7 millions vivent avec le VIH dont 200 000 nouveaux cas diagnostiqués en 2020 [4]. Au cours de cette infection, les atteintes rénales sont fréquentes et constituent un facteur de morbi-mortalité imposant des précautions particulières dans la prise en charge de ces patients [3]. Si l'avènement de traitements antirétroviraux hautement actifs (HAART) a révolutionné la survie des patients infectés par le VIH [3] en permettant de réduire les décès imputables au VIH par une élimination soutenue et durable du virus, il persiste cependant des atteintes rénales multiples glomérulaires, vasculaires et tubulo-interstitielles, parmi lesquelles on peut citer le syndrome d'infiltrat lymphocytaire diffus (DILS). Il s'agit d'une entité relativement rare qui survient chez les patients infectés par le VIH généralement mal contrôlés [3]. L'atteinte rénale n'est décrite que dans 6 à 8% des cas et se traduit par une néphropathie tubulo-interstitielle avec un infiltrat lymphocytaire de gravité variable. La prise en charge consiste en l'optimisation du traitement par HAART, associée le plus souvent à une corticothérapie, surtout en cas d'atteinte du système nerveux central et/ou d'atteinte rénale [1]

Nous rapportons un cas de DILS, découvert dans un contexte d'insuffisance rénale aiguë chez une patiente africaine, porteuse d'une infection par le VIH.

Le but de cette observation est de montrer l'efficacité du HAART sur les atteintes rénales du DILS sans recours à la corticothérapie.

## Observation

Il s'est agi d'une patiente âgée de 60 ans, née en Côte d'Ivoire, porteuse d'une infection par le VIH1 découverte en juillet 2016, au cours du bilan étiologique d'une anémie et d'une insuffisance rénale. Il n'y avait pas d'antécédents familiaux de néphropathie. Elle était naïve du traitement antirétroviral et ne présentait aucune affection opportuniste. Elle a consulté aux urgences du CHU de Yopougon en août 2016 pour un syndrome œdémato-ascitique, une anémie et une insuffisance rénale avec une créatinémie à 287 μmol/l.

**Figure 1:**
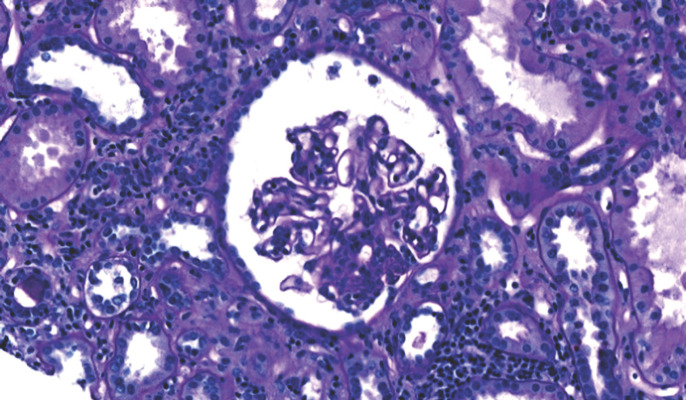
Hyalinose Segmentaire Focale avec infiltrat interstitiel important Focal Segmental Hyalinosis with significant interstitial infiltrate

**Figure 2:**
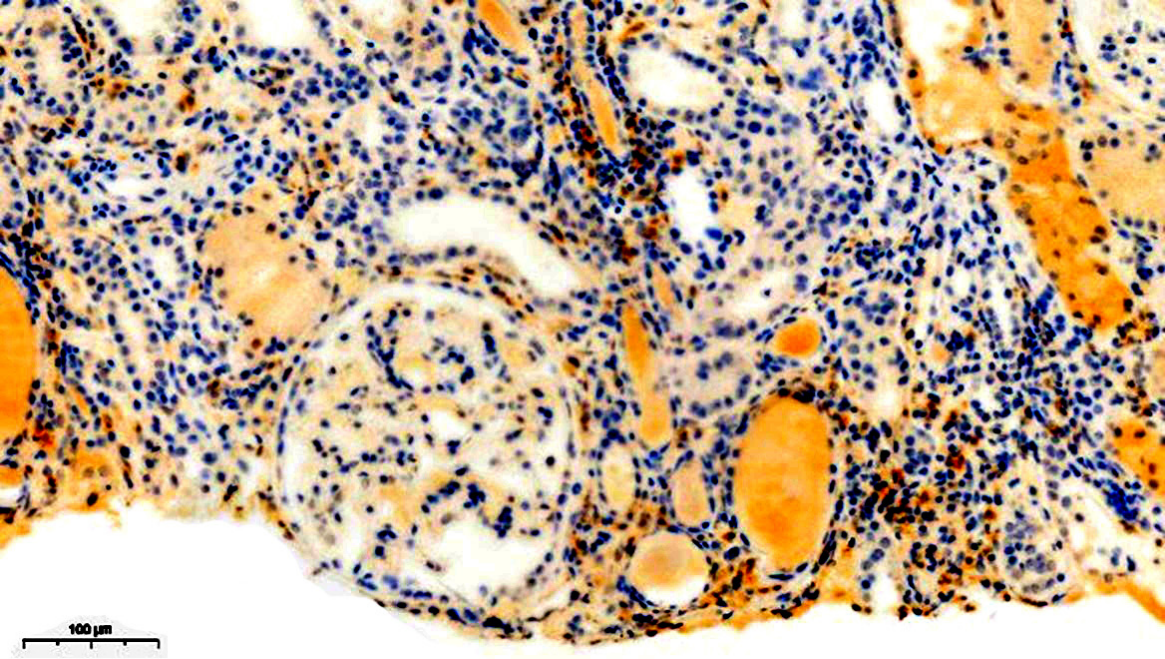
Biopsie montrant l'infiltrat de CD4 Biopsy showing CD4 infiltrate

**Figure 3:**
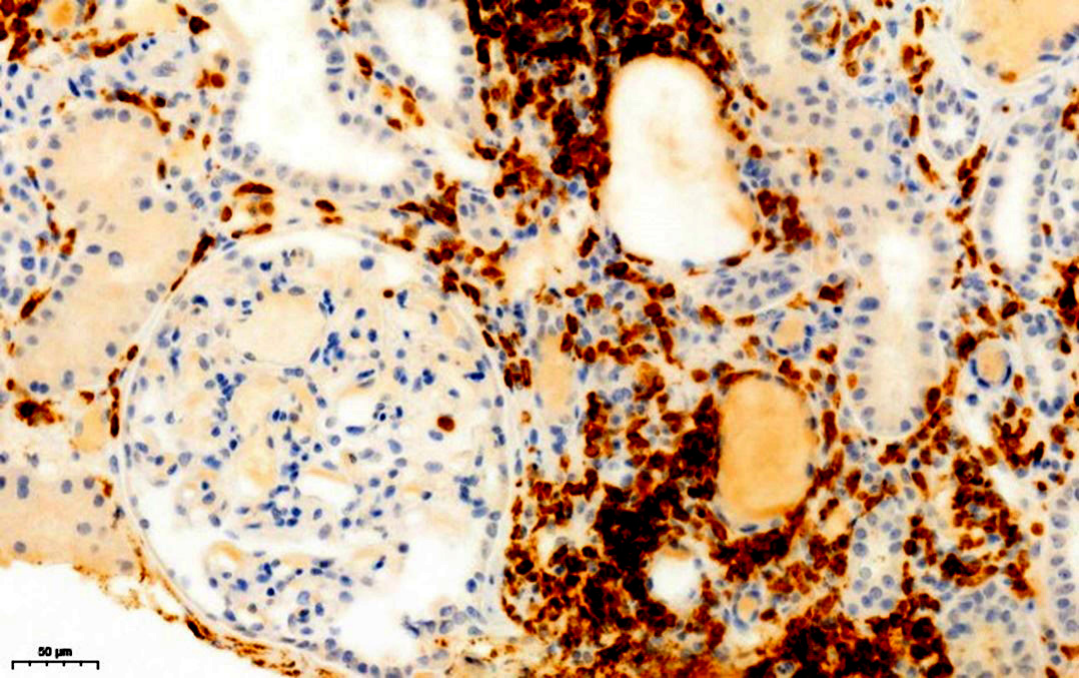
Biopsie montrant l'infiltrat important de CD8 Biopsy showing the important infiltrate of CD8

Sur le plan infectieux: le bilan de l'infection par le VIH a retrouvé un taux de CD4 à 30/mm^3^ et une charge virale à 1573, 3 copies soit 4, 72 log, la classant au stade C de l'OMS. L'exploration de l'insuffisance rénale a retrouvé un profil glomérulaire non néphrotique à 1 g/l de protéinurie, associée à une hypo-albuminémie disproportionnée à 9 g/l. La taille des reins était normale. L'histologie a mis en évidence la coexistence de quelques lésions de hyalinose segmentaire et focale (Fig. 1) avec un infiltrat interstitiel dense constitué majoritairement de lymphocytes CD8 définissant le DILS (Fig. 2 et Fig. 3). Les CD8 plasmatiques étaient à 1 593/µL, soit un rapport CD4/CD8 à 0, 18 avant le traitement par ARV.

Elle a présenté un syndrome sec buccal sans parotidomégalie. Il n'y avait pas d'atteinte pulmonaire en faveur du DILS. La tomodensitométrie par émission de positon (TEP) n'a pas retrouvé de processus évolutif médiastinal. Il n'y avait pas de granulome épithélioide dans l'interstitium et le bacille de Koch (BK) n'a été retrouvé dans aucun prélèvement, malgré les recherches approfondies.

La prise en charge a consisté à la mise sous traitements ARV par lamuvidine, abacavir et raltegravir.

L’évolution a été marquée par une amélioration de la fonction rénale avec une créatininémie passée de 287 μmol/l à 99 μmol/l en 3 mois, une régression de la protéinurie passant de 1 g/24h à 0, 24 g/24h, le taux de CD4 de 30 à 285/mm3 avec une charge virale VIH indétectable en 3 mois. Nous avons observé une normalisation du taux de lymphocytes TCD8 en 3 mois avec une amélioration du rapport CD4/CD8 à 0, 5. Compte-tenu de l'immunodépression et l'absence d'atteinte pulmonaire, il n'a pas été associé de corticothérapie.

## Discussion

### Épidémiologie

Le DILS est plus fréquent chez les Afro-américains (60%) que chez les autres Américains (26%) ou les Américains du Mexique (14%) [2]. Les données d'une étude américaine et d'une récente étude en Grèce ont rapporté une diminution de la fréquence du DILS, suggérant une probable efficacité des ARV [2]. La fréquence du DILS en Afrique au Sud du Sahara reste très mal connue.

### Signes cliniques

Le DILS est un syndrome de type Gougerot-Sjögren survenant chez les patients le plus souvent âgé de plus de 40 ans infectés par le VIH comme le cas de notre patiente [10]. Il se traduit par une hypertrophie bilatérale des glandes parotides, associée à un syndrome sec évoluant depuis plusieurs mois [10]; de nos jours, les symptômes respiratoires, hépatiques et rénaux sont rarement associés [10]. Le tableau clinique dans notre observation a associé un syndrome sec à une atteinte rénale isolée sans parotidomégalie, ni atteinte pulmonaire.

### Atteintes rénales

Bien que le DILS ait été depuis longtemps décrit dans la littérature, le premier cas d'atteinte rénale histologiquement prouvée n'a été rapporté qu'en 1990 [10]. Dans une étude portant sur 111 cas de DILS, l'atteinte rénale n’était retrouvée que dans 9% des cas [8].

Dans notre observation, nous décrivons l’évolution clinique et les caractéristiques histopathologiques de cette atteinte tubulo-interstitielle rare ou peut être sous-estimée. Plusieurs caractéristiques de l'atteinte rénale liées au DILS peuvent être observées. Selon la littérature [8] et dans notre observation, il s'agit d'une insuffisance rénale organique associée à une protéinurie tubulaire et des reins de grande taille traduisant une atteinte tubulo-interstitielle. Parmi les patients infectés par le VIH, un syndrome de lymphocytose CD8 avec infiltrat lymphocytaire multiviscéral semble plus fréquent chez les sujets de race noire positifs pour le gène HLA-DR5, peut-être parce qu'il existe un lien génétique déterminant la réponse immunitaire de l'hôte infecté par le VIH [5]. Par ailleurs, l'implication du gène HLA-A1B8DR3 dans la survenue du DISL a été décrite par Oksenhendler et al chez 3 sujets caucasiens infectés par le VIH [5]. Le même gène a été impliqué dans le même syndrome chez un patient gambien de 40 ans infecté par le VIH1 et qui a présenté une splénomégalie, hépatomégalie, lymphoadénopathie périphérique, parotidomégalie et chez lequel la biopsie rénale a montré une hyalinose segmentaire focale avec sclérose glomérulaire et un important infiltrat interstitiel de lymphocytes CD8; montant bien que l'expansion des lymphocytes T peut ne pas se limiter aux organes lymphoïdes [6].

Sur les biopsies rénales, les lésions du DILS sont caractérisées par une néphrite tubulo-interstitielle avec infiltrat interstitiel dense et irrégulier comprenant des lymphocytes et des monocytes. Une tubulite avec destruction tubulaire peut être associée parfois; les glomérules sont en général épargnés sauf dans des zones péri-capsulaires. L'immunophénotypage, montre que l'infiltrat cellulaire est composé principalement de LTCD3, LTCD8, mélangés avec des lymphocytes B et des histiocytes CD68 [10]. L'histologie dans notre observation montrait la coexistence de quelques lésions de hyaliniose segmentaire et focale, avec un infiltrat interstitiel dense, constitué majoritairement de lymphocytes TCD8 définissant le DILS. Par ailleurs la présence des lésions de hyalinose segmentaire et focale associées à l'infiltrat interstitiel peut faire aussi discuter une HIVAN (néphropathie associée au VIH). Classiquement la HIVAN constitue une complication tardive de l'infection survenant chez un patient VIH mal contrôlé dont le taux de CD4 est bas, la charge virale élevée et qui a des antécédents d'infections opportunistes [9]. Il existe cependant des formes précoces, voire diagnostiquées avant la positivité de la sérologie VIH ou survenant chez le patient VIH avant le stade sida et virologiquement bien contrôlé [7]. Le diagnostic de la HIVAN est histologique et se définit par l'association d'une forme particulière de hyalinose segmentaire et focale très sévère avec rétraction ou collapsus du floculus, d'une podocytose avec parfois des pseudo-croissants cellulaires, d'une dilatation microkystique des tubules et d'un œdème et d'une fibrose interstitiels associés à un infiltrat inflammatoire de lymphocytes T CD8. Dans notre observation il n'y avait cependant pas de collapsus du floculus.

### Traitement

Le DILS est considéré comme une lymphocytose à LTCD8 réactive à l'infection par le VIH: à la lumière des études selon lesquelles la prévalence du DILS aurait considérablement diminuée depuis l'introduction de thérapies antirétrovirales hautement actives, son traitement repose sur la combinaison des ARV aux corticoïdes [9]. Cette association peut être administrée simultanément ou peu de temps après une induction aux stéroïdes. Une faible corticothérapie peut être efficace dans le traitement de l'atteinte parotidienne, du syndrome de Gougerot-Sjögren; des doses plus élevées de corticoïdes sont requises lorsqu'il existe une atteinte des principaux organes pouvant mettre en jeu le pronostic vital du patient [7]. Les patients diagnostiqués à un stade précoce de la maladie et présentant des formes bénignes d'atteintes viscérales y compris rénales pourraient ne pas être traités par des stéroïdes et bénéficier d'un traitement ARV seul. Dans une série, 73% des patients porteurs de DILS avec les symptômes de Sicca ont reçu un traitement ARV seul [10]. Notre patiente n'a bénéficié que d'un traitement antirétroviral seul. Cependant Zafrani et al. à l'hôpital de Tenon ont observé une rechute clinique dans leur observation alors que la charge virale du VIH était indétectable dans le sang; montrant ainsi la disproportion entre le contrôle de la réplication du VIH et les symptômes du DILS [10].

### Évolution

Le DILS est un syndrome lymphoprolifératif bénin, mais qui peut cependant être la cause de graves dommages aux organes. Le pronostic est plutôt bon dans l'ensemble et le DILS est rarement cause de décès. Ces patients semblent avoir une progression lente vers le stade Sida. Ils ont moins d'infections opportunistes 6 à 27%, des taux de CD4 plus élevés et des taux faibles d'antigène P24 dans le sang périphérique par rapport aux patients infectés sans DILS [2]. Cela suggérerait le rôle protecteur de la lymphocytose CD8 [1].

L’évolution dans notre observation a été favorable avec une récupération de la fonction rénale (créatininémie à 99 μmol/l), une régression de la protéinurie, un taux de CD4 à 285/mm^3^, une charge virale VIH indétectable et une normalisation du taux de LTCD8 en 3 mois associée à une correction du rapport CD4/CD8 à 0, 5

## Conclusion

Le DILS est une affection rarement diagnostiquée en Afrique sub-saharienne, nous rapportons par cette observation un premier cas de DILS en COTE d'IVOIRE. Il survient le plus souvent dans un contexte de contrôle immuno-virologique insatisfaisant.

Les descriptions clinique et paraclinique de notre patiente permettront dans ce contexte africain d’évoquer le DILS devant une néphrite interstitielle aiguë chez les patients infectés par le VIH. Le syndrome sec, la lymphocytose à CD8 et les données de l'histologie rénale permettent d’établir le diagnostic de certitude et d'initier dans les meilleurs délais un traitement adéquat.

## Conflits D'intérêts

Les auteurs ne déclarent aucun conflit d'intérêt.

## References

[1] Ghrenassia E, Martis N, Boyer J, Burel-Vandenbos F, Mekinian A, Coppo P. (2015). The diffuse infiltrative lymphocytosis syndrome (DILS). A comprehensive review. J Autoimmun.

[2] Itescu S, Brancato LJ, Buxbaum J, Gregersen PK, Rizk CC, Croxson TS, Solomon GE, Winchester R. (1990). A diffuse infiltrative CD8 lymphocytosis syndrome in human immunodeficiency virus (HIV) infection: a host immune response associated with HLA-DR5. Ann Intern Med.

[3] Izzedine H ((2009)). Pathologies rénales au cours de l'infection par le VIH. La Lettre de l'Infectiologue.

[4] ONUSIDA Estimations épidémiologiques préliminaires de l'ONUSIDA (2021): statistiques mondiales sur le VIH. https://www.unaids.org.

[5] Oksenhendler E, Autran B, Gorochov G, D'Agay MF, Seligmann M, Clauvel JP. (1992). CD8 lymphocytosis and pseudotumoral splenomegaly in HIV infection. Lancet.

[6] Viard JP, Noël LH, Droz D, Bach JF (1992). A1B8DR3-associated CD8-positive T-cell expansion in HIV infection. Lancet.

[7] Winston JA, Bruggeman LA, Ross MD, Jacobson J, Ross L, D'Agati VD, Klotman PE, Klotman ME (2001). Nephropathy and establishment of a renal reservoir of HIV type 1 during primary infection. N Engl J Med.

[8] Winston JA, Burns GC, Klotman PE (1998). The human immunodeficiency virus (HIV) epidemic and HIV-associated nephropathy. Semin Nephrol.

[9] Winston JA, Klotman ME, Klotman PE (1999). HIV-associated nephropathy is a late not early manifestation of HIV-1 infection. Kidney Int.

[10] Zafrani L, Coppo P, Dettwiler S, Molinier-Frenkel V, Agbalika F, Guiard-Schmid JB, Pialoux G, Xu-Dubois YC, Rondeau E, Hertig A. (2007). Nephropathy associated with the diffuse infiltrative lymphocytosis syndrome. Kidney Int.

